# First Detection of Algal Caribbean Ciguatoxin in Amberjack Causing Ciguatera Poisoning in the Canary Islands (Spain)

**DOI:** 10.3390/toxins16040189

**Published:** 2024-04-13

**Authors:** Pablo Estevez, Juan Oses-Prieto, David Castro, Alejandro Penin, Alma Burlingame, Ana Gago-Martinez

**Affiliations:** 1 Department of Pharmaceutical Chemistry, University of California San Francisco, San Francisco, CA 94158, USA; paestevez@uvigo.es (P.E.); joses@cgl.ucsf.edu (J.O.-P.); alb@cgl.ucsf.edu (A.B.); 2 Biomedical Research Center (CINBIO), Department of Analytical and Food Chemistry, Campus Universitario de Vigo, University of Vigo, 36310 Vigo, Spain; dcastro@uvigo.es (D.C.); alpenin@alumnos.uvigo.es (A.P.)

**Keywords:** ciguatera poisoning, Caribbean CTX, CTX characterization, parallel reaction monitoring, fragmentation pathways

## Abstract

Ciguatera Poisoning (CP) is an illness associated with the consumption of fish contaminated with potent natural toxins found in the marine environment, commonly known as ciguatoxins (CTXs). The risk characterization of CP has become a worldwide concern due to the widespread expansion of these natural toxins. The identification of CTXs is hindered by the lack of commercially available reference materials. This limitation impedes progress in developing analytical tools and conducting toxicological studies essential for establishing regulatory levels for control. This study focuses on characterizing the CTX profile of an amberjack responsible for a recent CP case in the Canary Islands (Spain), located on the east Atlantic coast. The exceptional sensitivity offered by Capillary Liquid Chromatography coupled with High-Resolution Mass Spectrometry (cLC-HRMS) enabled the detection, for the first time in fish contaminated in the Canary Islands, of traces of an algal ciguatoxin recently identified in G. silvae and G. caribeaus from the Caribbean Sea. This algal toxin was structurally characterized by cLC-HRMS being initially identified as C-CTX5. The total toxin concentration of CTXs was eight times higher than the guidance level proposed by the Food and Drug Administration (0.1 ng C-CTX1/g fish tissue), with C-CTX1 and 17-hydroxy-C-CTX1 as major CTXs.

## 1. Introduction

Ciguatera Poisoning (CP) is among the most common seafood intoxications worldwide [1]. CP is mainly caused by the consumption of fish contaminated with ciguatoxins (CTXs). CTXs are a group of potent natural neurotoxins produced by dinoflagellates of the genera *Gambierdiscus* and *Fukuyoa* [2]. These toxins accumulate and metabolize in fish to a variety of toxic analogues which can cause poisoning in humans [3]. The symptomatology includes neurological, gastrointestinal and cardiovascular disorders and, to date, there is no effective treatment [4]. CP cases are mainly reported in tropical and subtropical areas of the world, such as the Caribbean Sea, the Indian Ocean, the Pacific Ocean and, more recently, in the east Atlantic Ocean [5,6]. Furthermore, some CP cases in Europe have been linked to imported fish from endemic regions of the Indian Ocean [7].

CTXs are lipophilic cyclic polyethers that are thermally stable and present at sub-ppb levels in fish [8]. Depending on their structure, CTXs can be classified as Caribbean, Indian or Pacific CTXs [5]. The availability of reference materials for these compounds is very scarce and mainly limited to research collaborations among scientists involved in the isolation of these toxins from natural sources, which makes the development of methods for their characterization very challenging. The most extended detection method for the identification of CTXs is Liquid Chromatography coupled to Mass Spectrometry (LC-MS) [9,10].

Most of the studies present in the literature have been focused on the Pacific CTXs [11,12,13]. These investigations have enabled the isolation and structural characterization of the majority of CTXs present in fish from these regions. Furthermore, they have facilitated the production of standards and the development of reliable detection methods, such as LC-MS/MS or even immunoassays [14,15]. This is in contrast with the research carried out in emerging regions such as the east Atlantic Ocean. CTXs were detected for the first time in this geographic area in the late 2000s and since then the European Food Safety Authority (EFSA) has been interested in obtaining not only occurrence data, but also the full toxin profile present in fish from this region [6,16,17]. Significant advances have been made during the last few years in the identification of the CTX profile by LC-MS in the east Atlantic Ocean [18,19,20].

However, the availability of contaminated samples related to CP is crucial for advancements not only in the identification of the CTXs involved in contamination but also in their isolation for subsequent chemical and toxicological evaluations. This progress enables the establishment of regulatory levels if necessary. The significant contribution of Capillary Liquid Chromatography to enhanced sensitivity in High-Resolution Mass Spectrometry detection (cLC-HRMS) has been demonstrated in a prior study carried out by the research team involved in this study [21]. In this study, the method was applied to characterize CTXs in a fish sample linked to a case of human intoxication with CP in the Canary Islands (Spain). The analysis revealed not only the presence of the main CTXs previously documented in this region but also, for the first time, the detection of an algal CTX recently identified in dinoflagellates and fish from the Caribbean Sea.

## 2. Results

**Figure 1:**
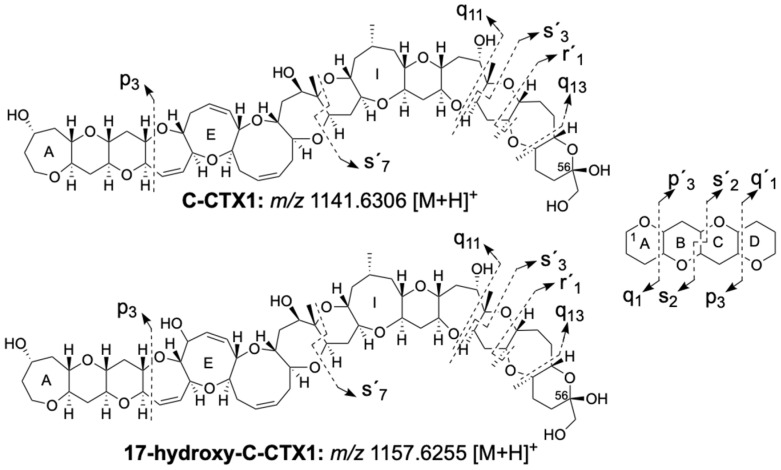
C-CTX1 and 17-hydroxy-C-CTX1 structures showing the main fragment ions detected in MS2 (labelled with arrows). The nomenclature used for the identification of the fragment ions was proposed in [22] and is summarized in the top right of the figure. P, q and s indicate the bonds which are cleaved, the subscript number is the number of rings contained in the fragment (intact ring, or ring fragment) and the prime symbol points out fragments towards the right end of the molecule.

The fish extract was analyzed by cLC-HRMS following the conditions described in [21]. MS-ddMS2 allows for the identification and confirmation of the toxins based on their ion pattern and exact mass ([M+H]^+^, [M+H−nH_2_O]^+^, [M + NH_4_]^+^, [M + Na]^+^ and [M + K]^+^). Additionally, the PRM mode was used to confirm the toxins based on their fragmentation. The nomenclature used for the identification of the fragment ions was proposed in [22] and is summarized in Figure 1. P, q and s indicate the bonds which are cleaved, the subscript number is the number of rings contained in the fragment (intact ring, or ring fragment) and the prime symbol points out fragments towards the right end of the molecule. The toxins were quantified with a calibration curve of C-CTX1 standard ranging from 0.61 to 20.00 ng/mL and each toxin was expressed in ng C-CTX1 equivalent/g fish tissue.

### 2.1. Ciguatoxin Profile

C-CTX1 was detected as the main toxin at a concentration of 0.46 ng C-CTX1 eq./g fish tissue. 17-hydroxy-C-CTX1 was also present at a concentration of 0.22 ng C-CTX1 eq./g fish tissue (Table 1). Both toxins were identified with traces of their respective 56-methoxy- congeners, 0.07 ng C-CTX1/g fish tissue for 56-methoxy-C-CTX1 and 0.04 ng C-CTX1/g fish tissue for 17-hydroxy-56-methoxy-C-CTX1 (Table 1). Additionally, traces of a new C-CTX algal analogue (C-CTX5), recently identified in [23] in algal from the Caribbean Sea, were also detected in the sample (Table 1). The total concentration of C-CTX1 eq. in the sample was 0.79 ng/g, which is around eight-fold above the guidance level proposed by the FDA (USA) for C-CTX1 [24].

**Figure 2:**
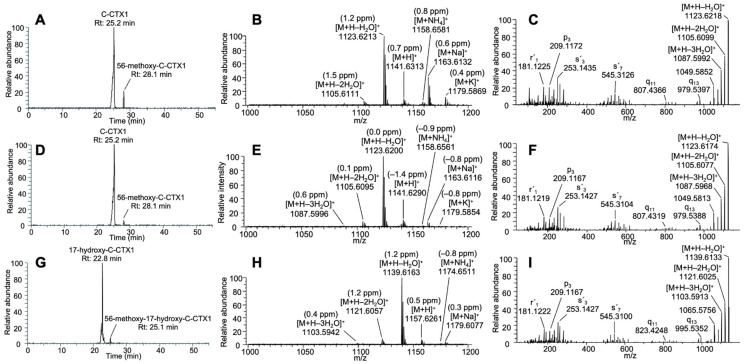
C-CTX1 standard: chromatogram with C-CTX1 at 25.2 min and traces of 56-methoxy-C-CTX1 at 28.1 min (**A**), full MS1 spectra showing the main molecular and pseudomolecular ions and their respective Δppm (**B**), and MS2 spectra selecting *m*/*z* 1123.6200 [M+H−H_2_O]^+^ as a precursor ion and applying a CE of 15 (**C**). C-CTX1 detected in the fish extract: chromatogram with C-CTX1 at 25.2 min and traces of 56-methoxy-C-CTX1 at 28.1 min (**D**), full MS1 spectra showing the main molecular and pseudomolecular ions and their respective Δppm (**E**), and MS2 spectra selecting *m*/*z* 1123.6200 [M+H−H_2_O]^+^ as a precursor ion and applying a CE of 15 (**F**). 17-hydroxy-C-CTX1 detected in the fish extract: chromatogram with 17-hydroxy-C-CTX1 at 22.8 min and traces of 17-hydroxy-56-methoxy-C-CTX1 at 25.1 min (**G**), full MS1 spectra showing the main molecular and pseudomolecular ions and their respective Δppm (**H**), and MS2 spectra selecting *m*/*z* 1139.6149 [M+H−H_2_O]^+^ as a precursor ion and applying a CE of 15 (**I**).

The C-CTX1 retention time (25.2 min), the ion pattern in MS1 and the MS2 fragmentation in the sample matched the C-CTX1 standard (Figure 2A–F, Tables S1 and S2). 17-hydroxy-C-CTX1 was detected at a retention time of 22.8 min with a prominent first water loss in MS1 at *m*/*z* 1139.6163 [M+H−H_2_O]^+^ (1.2 ppm) (Figure 2G,H). This compound was confirmed by its fragmentation in the PRM mode matching the data previously reported in [21] (Figure 1 and Figure 2I, Table S3).

### 2.2. Identification and Confirmation of C-CTX5

**Figure 3:**
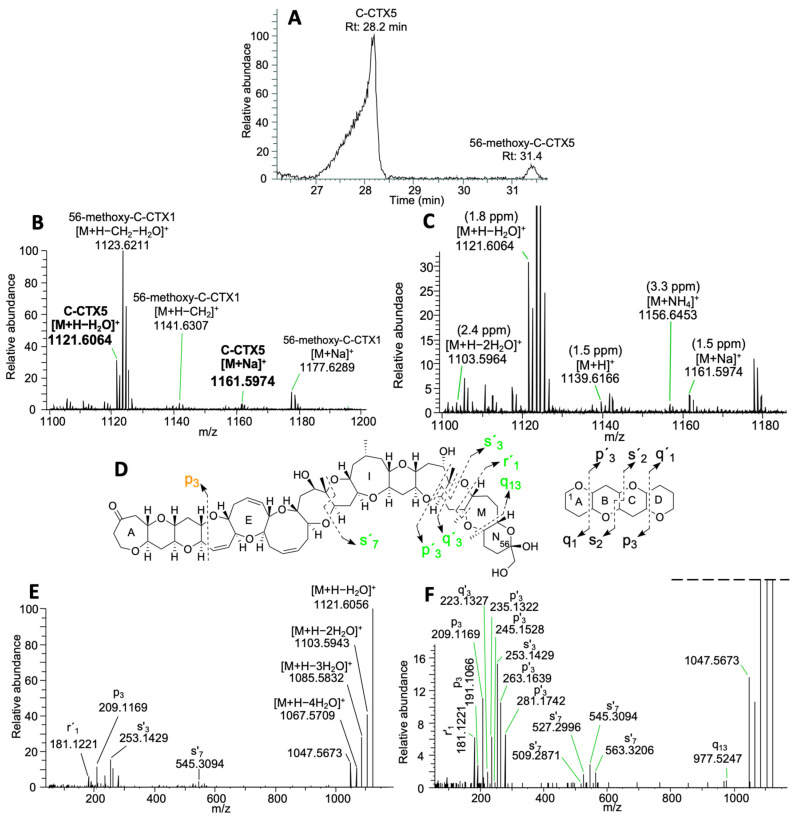
Detection of C-CTX5 in the fish extract from the Canary Islands (Spain). Chromatogram showing C-CTX5 at 28.19 min and its putative methoxy congener at 31.39 min (**A**); MS1 spectra of C-CTX5 (in bold) showing [M+H−H_2_O]^+^ and [M + Na]^+^ and its coelution with 56-methoxy-C-CTX1 (**B**); zoom-in of (**B**) showing C-CTX1 MS1 ions and their respective Δppm (**C**); C-CTX5 structure proposed in [22] showing the main fragment ions matching their data (in green) (**D**); MS2 spectra of C-CTX5 selecting *m*/*z* 1121.6043 [M+H−H_2_O]^+^ as a precursor ion at a CE of 15 (**E**); zoom-in of (**E**), “r” was used to represent fragmentations not included in the previous nomenclature proposed in [22] (**F**).

C-CTX5 was detected at 28.2 min with traces of its 56-methoxy analogue at 31.4 min (Figure 3A). C-CTX5 was eluted as a broad chromatographic peak, as detected for C-CTX1, resulting from the rapid on-column epimerization of the ketal in C-56 due to the acidic conditions [22]. C-CTX5 showed an ion pattern with a first water loss at *m*/*z* 1121.6064 [M+H−H_2_O]^+^ (1.8 ppm), sodium adduct at *m*/*z* 1161.5974 [M + Na]^+^ (1.5 ppm) and additional traces of ions such as [M+H]^+^, [M + NH_4_]^+^ and [M+H−2H_2_O]^+^ with Δppm < 3.5 (Figure 3B,C). This analogue coeluted with 56-methoxy-C-CTX1 (28.1 min) (Figure 1 and Figure 3B,C). PRM analyses selecting C-CTX5 first water loss as precursor ion *m*/*z* 1121.6043 [M+H−H_2_O]^+^ showed a similar fragmentation pathway to that described in [23]. Not only did the detection of successive water losses confirm C-CTX5, but it also confirmed the fragments described in [23] from fragmentation in the G-, and H-rings (s’7) and the K-, L- and M-rings (q13, s´7, s´3 and p´3) (Figure 3D–F, Table S4).

**Figure 4:**
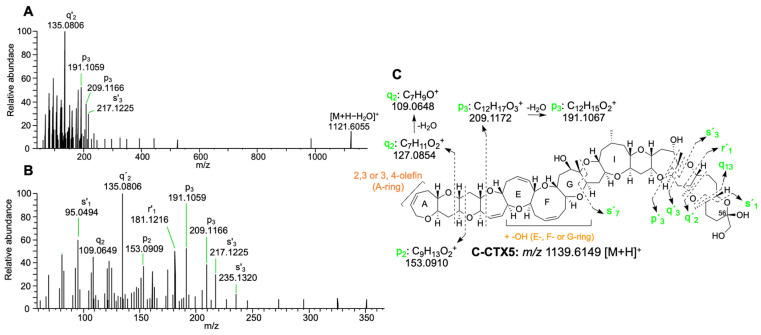
Structural characterization of C-CTX5. MS2 spectra selecting *m*/*z* 1121.6043 [M+H−H_2_O]^+^ as a precursor ion at a CE of 40 (**A**), zoom-in of (**A**) from *m*/*z* 50 to *m*/*z* 360 (**B**), proposal of the possible structure of C-CTX5 according to the MS2 fragmentation data (**C**).

Despite obtaining the same fragmentation pattern as that described in [23], the fragment at *m*/*z* 209.1171 (p3) from the fragmentation in the D-ring did not match the theoretical fragment of a 3-oxo metabolite and, consequently, additional PRM analyses at different CE levels were performed to corroborate the fragmentation of C-CTX5. The PRM analyses revealed that C-CTX5 should be a 2,3 or 3,4-olefing together with a hydroxylation in the E-, F- or G-rings instead of a 3-oxo metabolite of C-CTX1. The detection of fragments from the fragmentation in the B-, C- and D-rings (p3, q2 and p2) confirmed this possibility (Figure 4A–C, Table S5).

## 3. Discussion

As mentioned earlier, characterizing fish samples involved in human intoxications associated with Ciguatera Poisoning (CP) is crucial for advancing the risk assessment of CP in emerging regions such as the Canary Islands (Spain). This is particularly important because the evaluation of this risk in fish from official controls is not always efficient, given that the concentration of ciguatoxins (CTXs) in these samples might be limited.

The use of a sensitive method such as cLC-HRMS is a valuable approach for identifying and quantifying CTX analogues with both major and minor contributions to the overall CTX toxicity. The total CTX content of the sample analyzed in this study was 0.79 ng C-CTX1 eq./g, which should be considered a reasonably high CTX concentration to produce CP symptoms.

C-CTX1 (0.46 ng/g) and 17-hydroxy-C-CTX1 (0.22 ng/g) were present in concentrations clearly above the guidance level proposed by the FDA for C-CTX1 (0.1 ng/g) [24]. These results match the data in the literature in which, not only in the endemic areas of the Caribbean Sea, but also in the east Atlantic Ocean, C-CTX1 is always the main toxin present in the fish samples [19,25,26,27]. The presence of 56-methoxy- metabolites could be related to the artificial methoxylation of the CTXs during sample pretreatment [28].

C-CTX5, an algal ciguatoxin recently identified in [23] in *Gambierdisucs silvae* and *G. caribeaus* from the Caribbean Sea, was detected for the first time in fish from the east Atlantic Ocean. The detection of C-CTX5 in fish from this region could suggest that it might be a contributing toxin in *Gambierdiscus* strains from the area. Only taking into account the MS data, the structure of C-CTX5 matches a 2,3 or 3,4-olefing together with a hydroxylation in the E-, F- or G-rings instead of a 3-oxo metabolite of C-CTX1. Mudge et al., 2023, also proposed this possibility only using the MS data. However, the structure of C-CTX5 was proposed using selected fragments from MS2 and also the results after chemical and enzymatic conversions. C-CTX5 should be isolated in higher concentrations to investigate these discrepancies and study its MS fragmentation. Also, this would allow its structure to be elucidated by NMR. Unfortunately, the concentration of C-CTX5 in the fish sample analyzed in this study was below the LOQ, which impeded the obtention of better MS1 and MS2 spectra for structural characterization purposes. The low concentration of C-CTX5 also suggests that once transferred from *Gambierdiscus*, a metabolization process of C-CTX5 in fish might occur until it is converted into C-CTX1. A similar metabolization process has been reported for some Pacific CTXs (P-CTXs) [3].

## 4. Conclusions

The fish sample analyzed in this study, which was consumed and linked to an outbreak of CP in the Canary Islands (Spain), was successfully characterized using a sensitive method involving Capillary Liquid Chromatography coupled to High-Resolution Mass Spectrometry (cLC-HRMS). The concentration of CTXs, expressed in C-CTX1 eq., was eight-fold above the guidance level proposed by the FDA, with C-CTX1 being the major toxin followed by 17-hydroxy-C-CTX1. Traces of putative C-CTX5 (an algal toxin) were detected for the first time in fish from the east Atlantic Ocean, suggesting that C-CTX5 might be a precursor to C-CTX1 in *Gambierdiscus* strains from this region. MS1 and MS2 data showed that this compound might be a 2,3 or 3,4-olefin together with a hydroxylation in the E-, F- or G-rings instead of a 3-oxo metabolite, as initially proposed. However, the low toxin amount in the sample and the absence of an authentic C-CTX5 standard make the characterization of its structure challenging. Further isolation of this compound in higher concentrations from fish or dinoflagellates would enable its complete structural characterization by NMR and confirm its identity as C-CTX5.

## 5. Materials and Methods

### 5.1. Standard and Sample

C-CTX1 standard (20 ng) was kindly provided by Dr. Robert W. Dickey (previously U.S. Food and Drug Administration) [29].

The fish sample consisted of a raw portion of amberjack fish (*Seriola* sp.) tissue captured in the spring of 2023 in Fuerteventura in the Canary Islands (Spain). This fish sample was consumed and linked to an outbreak of ciguatera and was kindly provided by the Canary Islands Government Health Services through the Instituto Universitario de Sanidad Animal y Seguridad Alimentaria (IUSA) from the University of Las Palmas de Gran Canaria (ULPGC) on the course of the activities of the EuroCigua II project (GP/EFSA/KNOW/2022/03).

### 5.2. Sample Preparation and cLC-HRMS Analyses

Sample pretreatment and cLC-HRMS analyses were performed as described in [21].

## Figures and Tables

**Table 1:** Concentration expressed in ng C-CTX1 eq./g fish tissue and retention time of each CTX analogue detected in the fish extract from the Canary Islands (Spain) by cLC-HRMS.

Method	Toxin	ng C-CTX1 eq./g Fish Tissue	Retention Time (min)
cLC-HRMS	C-CTX1	0.46	25.2
	56-methoxy-C-CTX1	0.07	28.1
	17-hydroxy-C-CTX1	0.22	22.8
	17-hydroxy-56-methoxy-C-CTX1	0.04	25.1
	C-CTX5	<LOQ	28.2
	56-methoxy-C-CTX5	<LOQ	31.4
	Σ (C-CTXs)	0.79	

## Data Availability

Data available on request.

## Supplementary Materials

The following supporting information can be downloaded at: https://www.mdpi.com/article/10.3390/toxins16040189/s1, Table S1. Mass error of C-CTX1 fragments after PRM analyses of C-CTX1 standard (20 ng/mL) selecting *m*/*z* 1123.6200 [M+H–H_2_O]^+^ as a precursor ion at a CE of 15; Table S2. Mass error of C-CTX1 fragments after PRM analyses of C-CTX1 in amberjack sample selecting *m*/*z* 1123.6200 [M+H–H_2_O]^+^ as a precursor ion at a CE of 15; Table S3. Mass error of 17-hydroxy-C-CTX1 fragments after PRM analyses of 17-hydroxy-C-CTX1 in amberjack sample selecting *m*/*z* 1139.6149 [M+H–H_2_O]^+^ as a precursor ion at a CE of 15; Table S4. Mass error of C-CTX5 fragments after PRM analyses of C-CTX5 in amberjack sample selecting *m*/*z* 1121.6043 [M+H–H_2_O]^+^ as a precursor ion at a CE of 15; Table S5. Mass error of C-CTX5 fragments after PRM analyses of C-CTX5 in amberjack sample selecting *m*/*z* 1121.6043 [M+H–H_2_O]^+^ as a precursor ion at a CE of 40.
