# Supplementary material for: First Detection of Algal Caribbean Ciguatoxin in Amberjack Causing Ciguatera Poisoning in the Canary Islands (Spain)
Source: Toxins (Basel). 2024 Apr 13;16(4):189. doi: 10.3390/toxins16040189 (PMC11054928; doi:10.3390/toxins16040189)
Supplement: Supplementary file 1 [file toxins-16-00189-s001.zip › toxins-2935332-supplementary.pdf]

Supplementary Materials: First Detection of Algal Caribbean Ciguatoxin in Amberjack Causing Ciguatera Poisoning in the Canary Islands (Spain)

Pablo Estevez, Juan Oses-Prieto, David Castro, Alejandro Penin, Alma Burlingame and Ana Gago-Martinez

Table S1. Mass error of C-CTX1 fragments after PRM analyses of C-CTX1 standard (20 ng/mL) selecting *m*/*z* 1123.6200 $[M+H–H_2O]^+$ as a precursor ion at a CE of 15.

Ion	Molecular Formula	Theoretical *m*/*z*	Measured *m*/*z*	Error (ppm)
$[M+H-H_2O]^+$	$C_{62}H_{91}O_{18}^+$	1123.6200	1123.6218	1.6
$[M+H-2H_2O]^+$	$C_{62}H_{89}O_{17}^+$	1105.6094	1105.6099	0.5
$[M+H-3H_2O]^+$	$C_{62}H_{87}O_{16}^+$	1087.5989	1087.5992	0.3
q_{13}	$C_{55}H_{79}O_{15}^+$	979.5414	979.5397	-1.7
q_{11}	$C_{46}H_{63}O_{12}^+$	807.4314	807.4366	6.4
s'_7	$C_{31}H_{45}O_8^+$	545.3109	545.3126	3.1
s'_3	$C_{14}H_{21}O_4^+$	253.1434	253.1435	0.4
p_3	$C_{12}H_{17}O_3^+$	209.1172	209.1172	0.0
r'_1	$C_{11}H_{17}O_2^+$	181.1223	181.1225	1.1

Table S2. Mass error of C-CTX1 fragments after PRM analyses of C-CTX1 in amberjack sample selecting *m*/*z* 1123.6200 $[M+H–H_2O]^+$ as a precursor ion at a CE of 15.

Ion	Molecular Formula	Theoretical *m*/*z*	Measured *m*/*z*	Error (ppm)
$[M+H-H_2O]^+$	$C_{62}H_{91}O_{18}^+$	1123.6200	1123.6174	-2.3
$[M+H-2H_2O]^+$	$C_{62}H_{89}O_{17}^+$	1105.6094	1105.6077	-1.5
$[M+H-3H_2O]^+$	$C_{62}H_{87}O_{16}^+$	1087.5989	1087.5968	-1.9
q_{13}	$C_{55}H_{79}O_{15}^+$	979.5414	979.5388	-2.7
q_{11}	$C_{46}H_{63}O_{12}^+$	807.4314	807.4319	0.6
s'_7	$C_{31}H_{45}O_8^+$	545.3109	545.3104	-0.9
s'_3	$C_{14}H_{21}O_4^+$	253.1434	253.1427	-2.8
p_3	$C_{12}H_{17}O_3^+$	209.1172	209.1167	-2.4
r'_1	$C_{11}H_{17}O_2^+$	181.1223	181.1219	-2.2

Table S3. Mass error of 17-hydroxy-C-CTX1 fragments after PRM analyses of 17-hydroxy-C-CTX1 in amberjack sample selecting *m/z* 1139.6149 $[M+H–H_2O]^+$ as a precursor ion at a CE of 15.

Ion	Molecular Formula	Theoretical *m/z*	Measured *m/z*	Error (ppm)
$[M+H−H_2O]^+$	$C_{62}H_{91}O_{19}^+$	1139.6149	1139.6133	-1.4
$[M+H−2H_2O]^+$	$C_{62}H_{89}O_{18}^+$	1121.6043	1121.6025	-1.6
$[M+H−3H_2O]^+$	$C_{62}H_{87}O_{17}^+$	1103.5938	1103.5913	-2.2
q_{13}	$C_{55}H_{79}O_{16}^+$	995.5363	995.5352	-1.1
q_{11}	$C_{46}H_{63}O_{13}^+$	823.4263	823.4248	-1.8
s'_7	$C_{31}H_{45}O_8^+$	545.3109	545.3100	-1.7
s'_3	$C_{14}H_{21}O_4^+$	253.1434	253.1427	-2.8
p_3	$C_{12}H_{17}O_3^+$	209.1172	209.1167	-2.4
r'_1	$C_{11}H_{17}O_2^+$	181.1223	181.1222	-0.6

Table S4. Mass error of C-CTX5 fragments after PRM analyses of C-CTX5 in amberjack sample selecting *m/z* 1121.6043 $[M+H-H_2O]^+$ as a precursor ion at a CE of 15.

Ion	Molecular Formula	Theoretical *m/z*	Measured *m/z*	Error (ppm)
$[M+H-H_2O]^+$	$C_{62}H_{89}O_{18}^+$	1121.60434	1121.6056	1.1
$[M+H-2H_2O]^+$	$C_{62}H_{87}O_{17}^+$	1103.59378	1103.5943	0.5
$[M+H-3H_2O]^+$	$C_{62}H_{85}O_{16}^+$	1085.58321	1085.5832	0.0
$[M+H-4H_2O]^+$	$C_{62}H_{83}O_{15}^+$	1067.57265	1067.5709	-1.6
$[M+H-5H_2O]^+$	$C_{62}H_{81}O_{14}^+$	1049.56208	1049.5566	-5.2
q_{13}	$C_{55}H_{79}O_{15}^+$	977.5257	977.5247	-1.0
s'_7	$C_{31}H_{47}O_9^+$	563.3215	563.3206	-1.6
s'_7	$C_{31}H_{45}O_8^+$	545.3109	545.3094	-2.8
s'_7	$C_{31}H_{43}O_7^+$	527.3003	527.2996	-1.3
s'_7	$C_{31}H_{41}O_6^+$	509.2898	509.2871	-5.3
p'_3	$C_{16}H_{25}O_4^+$	281.1747	281.1742	-1.8
p'_3	$C_{16}H_{23}O_3^+$	263.1642	263.1639	-1.1
s'_3	$C_{14}H_{21}O_4^+$	253.1434	253.1429	-2.0
p'_3	$C_{16}H_{21}O_2^+$	245.1536	245.1528	-3.3
s'_3	$C_{14}H_{19}O_3^+$	235.1329	235.1322	-3.0
q'_3	$C_{13}H_{19}O_3^+$	223.1329	223.1327	-0.9
p_3	$C_{12}H_{17}O_3^+$	209.1172	209.1169	-1.4
p_3	$C_{12}H_{15}O_2^+$	191.1067	191.1066	-0.5
r'_1	$C_{11}H_{17}O_2^+$	181.1223	181.1221	-1.1

Table S5. Mass error of C-CTX5 fragments after PRM analyses of C-CTX5 in amberjack sample selecting *m/z* 1121.6043 $[M+H–H_2O]^+$ as a precursor ion at a CE of 40.

Ion	Molecular Formula	Theoretical *m/z*	Measured *m/z*	Error (ppm)
s'_3	$C_{14}H_{19}O_3^+$	235.1329	235.1320	-3.8
s'_3	$C_{14}H_{17}O_2^+$	217.1223	217.1225	0.9
p_3	$C_{12}H_{17}O_3^+$	209.1172	209.1166	-2.9
p_3	$C_{12}H_{15}O_2^+$	191.1067	191.1059	-4.2
r'_1	$C_{11}H_{17}O_2^+$	181.1223	181.1216	-3.9
p_2	$C_9H_{13}O_2^+$	153.0910	153.0909	-0.7
q'_2	$C_9H_{11}O^+$	135.0804	135.0806	1.5
q_2	$C_7H_9O^+$	109.0648	109.0649	0.9
s'_1	$C_6H_7O^+$	95.0491	95.0494	3.2
